# Sculptors of African Women’s Bodies: Forces Reshaping the Embodiment of Female Genital Cutting in the West

**DOI:** 10.1007/s10508-020-01710-1

**Published:** 2020-04-23

**Authors:** Crista E. Johnson-Agbakwu, Emily Manin

**Affiliations:** 1 Southwest Interdisciplinary Research Center, Watts College of Public Service and Community Solutions, Arizona State University, MC 5120, 201 North Central Avenue, 33rd Floor, Phoenix, AZ 85004 USA; 2 Refugee Women’s Health Clinic, Obstetrics and Gynecology, Valleywise Health, Phoenix, AZ USA

Female genital cutting (FGC) continues to garner increasing attention across western health-care systems seeking to optimize care and outcomes among migrant communities affected by this practice. Besides clinical knowledge gaps, health-care providers may also lack an understanding of the historical, sociocultural, and geopolitical forces that undergird women’s health-seeking behavior, experiences with care, and decision making through the lens of cultural relativism (Evans et al., 2019a, b; Hess, Weinland, & Saalinger, 2010; Kaplan, Hechavarría, Bernal, & Bonhoure, 2013; Lane, Johnson-Agbakwu, Warren, Budhathoki, & Cole, 2019; Lazar, Johnson-Agbakwu, Davis, & Shipp, 2013; Smith & Stein, 2017; Sureshkumar et al., 2016; Tsianakas & Liamputtong, 2002). The Target Article by Brady, Connor, Chaisson, Sharif Mohamed, and Robinson (2019) presents a conceptual model informed by the theory of planned behavior (TPB), which guides providers on how to engage in culturally sensitive discussions and shared decision making on FGC-related care with patients, their partners, and their families. In this Commentary, we highlight the flux of migratory and acculturative processes and the prevalent hegemonic discourse which continually reshapes the embodiment of FGC and may implicitly influence women’s decision making regarding defibulation management.

## Attitudes, Norms, and Control in Flux

Acculturation is a process of social, psychological, and cultural adaptation that takes place over a period of time as differing cultural groups adjust to the prevailing cultural norms of a given society (Rudmin, 2003). It also considers how cross-border transnational social networks reinforce cultural norms from communities of origin (Berry, 2013; Mouw, Edelblute, Verdery, & Chavez, 2014; Salant & Lauderdale, 2003; Vacca, Solano, Lubbers, Molina, & McCarty, 2018). In the context of migration among FGC-affected populations, proxies of acculturation have typically relied on such factors as English fluency, language preference, age of first arrival, and length of time in the host nation. Multi-dimensional scales are increasingly being developed to capture the extent to which migrants may concomitantly derive their identity from both their country of origin and their host country (Abraído-Lanza, Armbrister, Flórez, & Aguirre, 2006; Agbemenu, 2016; Berry, 2005). Research is only beginning to elucidate how the acculturative experience may impact utilization of health-care services and disparities in health outcomes using context-dependent and validated metrics of acculturation (Johnson-Agbakwu, Flynn, Asiedu, Hedberg, & Breitkopf, 2016; Michlig, Mackey, & Johnson-Agbakwu, 2020).

The fluctuation of beliefs and behaviors inherent in acculturation should be considered in conjunction with the TPB, which posits that intention to engage in a behavior is based on one’s attitudes, perceived norms, and perceived autonomy with respect to this behavior (Ajzen, 1991). The convention of FGC and its sociocultural norms evolves in the context of migration (Chiatti, 2019; Gele, Johansen, & Sundby, 2012; Johnsdotter & Essén, 2016; Vangen, Johansen, Sundby, Træen, & Stray-Pedersen, 2004; Wahlberg, Johnsdotter, Selling, Källestål, & Essén, 2017). Coined by Berg and Denison (2013) as “a tradition in transition,” global migration from countries where FGC is highly prevalent to those where the practice is uncommon, criminalized, and stigmatized precipitates a cultural shift in attitudes and decline in support (see also Johnsdotter, 2018; Johnsdotter & Essén, 2016). Among diasporic communities, the underpinning forces that perpetuate (cultural tradition, sexual morals, marriageability, religion, aesthetics, perceived health benefits, and male sexual enjoyment) and hinder (health consequences, lack of religious requirement, illegality, male sexual needs, and lack of support from males) the continuance of FGC are subjected to cross-cultural scrutiny and pressures that prompt a reconsideration of the practice (Berg & Denison, 2013). While a cross-sectional study in Norway suggests that a shift in beliefs generally occurs after > 4 years in the west (Gele, Sagbakken, & Kumar, 2015), the fluidity of shifting attitudes toward FGC in conjunction with attempts to preserve sociocultural norms amidst an increasingly hostile geopolitical climate toward migrants suggests that acculturation is not at all linear (Abraído-Lanza et al., 2006; Bewley, Creighton, & Momoh, 2010; Shirazi, 2017; Wahlberg et al., 2017; Young, 2020). A qualitative study in Norway found that while Somali and Sudanese migrants possessed negative attitudes toward infibulation due to its inherent health risks, they also opposed premarital defibulation (Johansen, 2017).

When considering FGC-affected women’s decision-making processes regarding intention to pursue vulvar reconstructive procedures, it is critical to understand how attitudes toward FGC, perceived norms about the practice, and autonomy in decision making may be influenced by such acculturative processes and the maintenance of social networks both within diasporic host communities and communities of origin. Furthermore, understanding how a migrant woman’s personal perspectives may be influenced by social interactions and interpersonal relationships (both locally and abroad) may shed light on their motivations to seek care and their autonomy in decision making regarding FGC-related care. Koukoui, Hassan, and Guzder (2017) posited that even geographic distance brought about by displacement does not shield diasporic women from matriarchal pressures, namely by female elders. However, the dynamic acculturative forces affecting views of FGC also augment migrant women’s control over decision making and husbands’ capacities to openly oppose FGC. In the process of relinquishing sociocentric norms in favor of more egocentric western values, the extended family may carry less weight in decisions surrounding infibulation (Essén et al., 2000; Johnson-Agbakwu, Helm, Killawi & Padela, 2014). Needless to say, such complex dynamics may not change predictably with acculturation. Consequently, when interacting with patients and their families, providers should be sensitive to and acknowledge these forces.

## The “Othering” of African Women’s Bodies, Genital Self-Image, and Iatrogenic Pathologization

Upon arriving in the west, migrant women with FGC immediately encounter the “othering” of those who do not uphold dominant societal “norms” (Landry, 2018). Linguistic accents, scents, hairstyles and/or head coverings, attire, cultural norms, religious practices, and FGC status further accentuate this phenomenon. While the practice of FGC is not limited to the African sub-continent, being rather widespread throughout regions of Southeast Asia and the Middle East, special attention must be paid to the influence of racism and racial/ethnic stereotypes on the pervasive “othering” of African migrants with FGC in the West.

For health-care providers to better understand this phenomenon experienced by a large subset of their patients with FGC, Black African women, they must consider the historical traumas of slavery, the exploitation of Sarah Baartman across 19th century Europe as the “Hottentot Venus,” and other racialized stereotypes of Black women’s bodies (Koukoui, 2019; Magubane, 2001; West, 1995). Such stereotypes appeared as recently as 2012 when a YouTube video,,^1^,^2^,^3^,^4^ from Swedish World Art Day went viral, depicting a performance artist whose head was transformed into a 19th century minstrel blackface caricature and emblazoned on a bare-chested cake of a Black woman’s torso. The “cake” screams out in agony each time the Swedish Minister of Culture cuts a slice of the “woman” undergoing faux female genital mutilation. To add further insult to injury, the Minister occasionally feeds the very cake to the ailing “woman” (unwittingly effectuating a war crime through autocannibalism); sensationalizing and reinforcing the stereotype of the “barbaric” African amidst a surrounding sea of White faces who are reveling, snapping photographs, and eating the very “mutilation cake” (Fawcett, 2014). Such a depiction illustrates how the hegemonic discourse around FGC can swiftly exert deleterious impact, whether overtly or implicitly, on women’s embodied experiences living with FGC in the West.

While it is important to acknowledge the myriad harms of FGC, it should not be assumed that all women experience harms, the extent and manifestation of which must be considered against the backdrop of exposure to other past traumas and victimization resulting from war, forced displacement, rape, forced marriage, and domestic violence among diasporic populations (Fox & Johnson-Agbakwu, 2020). A holistic lens is necessary to consider the full range of factors which may impact sexual function in FGC-affected women without further stigmatizing them by only narrowly focusing on the state of their altered genitalia (Sharif Mohamed, Wild, Earp, Johnson-Agbakwu, & Abdulcadir, 2020). Furthermore, it is also critically important to distinguish the ethical debate surrounding genital alterations performed on minors, and consider the human right to bodily integrity and genital autonomy of all minors regardless of gender (Brussels Collaboration on Bodily Integrity, 2019; Earp, 2016b, 2020). This must be further contextualized by the sociopolitical discourse around gender and race which are pivotal to the development of truly unbiased policy (Atallah et al., 2016), failing to do so further “others” non-White migrant women’s bodies (Baillot, Murray, Connelly, & Howard, 2018; Johansen, 2017).

Some diasporic women with FGC may maintain the dominant view from their homeland of FGC being beautiful, pure, hygienic, symbolically meaningful, and enhancing one’s femininity (Ahmadu, Shweder, & Richard, 2009; Chalmers, Med, & Hashi, 2000; Johnsdotter, 2018; Londoño Sulkin, 2016; Sharif Mohamed et al., 2020). While large-scale empirical evidence is lacking, several qualitative studies have described women being disconcerted and unsettled by their genital appearance post-defibulation, feeling “abnormal,” “too open,” “exposed,” or “like an empty space” (Abdulcadir, Margairaz, Boulvain, & Irion, 2011; Abdulcadir, McLaren, Boulvain, & Irion, 2016b; Chalmers et al., 2000; Johnson-Agbakwu & Warren, 2017; Moxey & Jones, 2016; Smith & Stein, 2017). Many of these women are conflicted between function and aesthetics, between their ability to now engage in penile–vaginal intercourse without pain, yet experience distress in their postsurgical vulvar appearance (Evans et al., 2019b; Safari, 2013). Such cognitive dissonance is augmented by the realization that while they may place supreme value on upholding cultural mores and aesthetics, their partners and providers may prioritize functional outcomes (Evans et al., 2019b; Safari, 2013).

Discomfort with genital appearance post-defibulation is further complicated by the fact that re-infibulation is criminalized throughout much of the west. In those nations where legislation does not explicitly outlaw re-infibulation, it is strongly denounced by professional organizations who admonish providers to refuse such patient requests, considering it a form of “medicalized female genital mutilation”; while concomitantly oblivious to the moral double-standard that condones female genital cosmetic surgery (FGCS) among consenting, wealthy women (Ahmadu, 2017; Baillot et al., 2018; Earp, 2016a; Johansen, Ziyada, Shell-Duncan, Kaplan, & Leyed, 2018; Perron, Senikas, Burnett, & Davis, 2020), despite the lack of long-term safety and efficacy data (Barbara et al., 2017; Magon & Alinsod, 2017; Perron et al., 2020; Serati, Salvatore, & Rizk, 2018). Since both re-infibulation and FGCS are primarily performed on consenting adults, we argue that “partial” re-infibulation/“partial” defibulation^5^ should be reconsidered as FGCS rather than FGC among women with otherwise healthy sexual function and autonomy in requesting vulvar reconstructive procedures (Shahvisi & Earp, 2019). Emerging evidence has delineated the general preservation of sexual erectile tissues important for sexual arousal, orgasm, and pleasure among women with Type IIIb FGC (Abdulcadir et al., 2016a; Nour, Michels, & Bryant, 2006), dispelling the sweeping, generalized myth that FGC completely disrupts a woman’s capacity for sexual enjoyment, which, in and of itself, can produce harm (Johnsdotter, 2018).

Within the context of migration, ubiquitous stigmatization, stereotyping, “othering,” and body-shaming prevalent in public sociopolitical FGC discourse may engender an iatrogenic pathologization that negatively affects the genital self-image, sexual self-esteem, and sexual function of FGC-affected women (Brussels Collaboration on Bodily Integrity, 2019; Earp, 2020; Johnsdotter, 2018; Johnsdotter & Essén, 2016; Johnson-Agbakwu & Warren, 2017; Koukoui, 2019; Sharif Mohamed et al., 2020). Adolescents exploring their sexuality are particularly subject to this phenomenon, especially when engaging in relationships with partners outside of their own ethnocultural identity (Johnson-Agbakwu & Warren, 2017; Perron et al., 2020). The cultural encoding of FGC is context-specific, with supportive sociocultural protections being lost once displaced from one’s homeland, creating traumatic stress and vulnerabilities both within and outside of the healthcare setting (Koukoui, 2019; Lien & Schultz, 2013; Schultz & Lien, 2014).

It is the trauma that arises years later through migration, protracted displacement, when a woman is informed of the negative biomedical and sexual consequences of FGC. Diasporic women with FGC migrate from a land where the practice grants legitimacy, status, and respect, to a country where it is regarded as an egregious violation of human rights and where sanctions against FGC are more stringent. The myriad information on its deleterious effects compels women to revisit their experience. From an etiological perspective, this new perception of FGC can trigger distress and at times the reification of trauma. (Koukoui, 2019, p. 104)

The power hierarchies displayed in the manner in which health-care providers discuss FGC and surgical reconstructive options with patients may impact their health-seeking behavior and decision making (Johnson-Agbakwu & Warren, 2017). Research has documented providers’ pathologizing terminology such as “mutilation” and/or statements of “disfigurement,” narrow focus on the state of the genitalia while overlooking the primary reason(s) for the health visit, non-verbal bodily cues of shock and horror, patronizing overtones, and the parade of trainees and other health-care providers to gaze upon the clinical case of the altered genital anatomy (Evans et al., 2019b; Perron et al., 2020; Scamell & Ghumman, 2019; Turkamani, Homer, & Dawson, 2019). One patient with FGC said of her gynecological exam: “All of them just wanted to look at me. I didn’t understand why and nobody asked me,” while another added, “My genitals were on display. A group of white-coated staff [came] and [looked] and [talked] to each other with disgust” (Scamell & Ghumman, 2019). Such experiences can generate feelings of inadequacy, discomfort, shame and anger, and are particularly impactful in light of inherent patient–provider power dynamics amidst engrained societal racial and cultural hegemony (Johnsdotter, 2018; Koukoui, 2019). Providers should be mindful of these dynamics before engaging their patients as teaching spectacles, and must be very intentional and sensitive to the language and tone used in their interactions with their patients (Perron et al., 2020).

In tandem, the demand for clitoral reconstructive procedures have burgeoned in recent years, despite the lack of long-term data on safety and efficacy amidst an array of complex ethical, psychosocial, physiological, and cultural conundrums (Sharif Mohamed et al., 2020). Some proponents encourage women to “break with a culture that oppresses women,” basing their patients’ candidacy for surgery on their “maturity” and desire to “become a free woman,” rather than appropriately outlining the risks, benefits, clinical indications for and anatomic expectations of surgical intervention, and potential alternatives, after psychosexual counseling and education (Villani, 2009). Until the media, policymakers, health-care systems, and providers adopt compassionate, culturally relativistic, and nonjudgmental approaches, incorporating language that avoids “othering” migrant women with FGC, patients will continue to grapple with the iatrogenic pathologization that occurs in their experiences with care in the west.

## Social Determinants of Health and Their Influence on Distrust and Health-Seeking Behavior

Nationalistic, xenophobic, anti-migrant, and anti-Muslim rhetoric have swept across much of the geopolitical discourse throughout the west (Shirazi, 2017; Young, 2020). Against this backdrop, FGC-affected communities have been under siege with threats of deportation, airport searches, national news headline-garnering criminal investigations (Bootwala, 2019), and fears of daughters being taken away for perceived risk of having undergone FGC (Costello, 2015; Essén & Johnsdotter, 2004; Johansen et al., 2018). As survivors of human rights atrocities and the victimization of war and conflict, they are further plunged deeper into the shadows as “hidden” migrant communities, underutilizing health-care services (Fox & Johnson-Agbakwu, 2020) and stymying acculturative processes (Berry, 1997; Michlig et al., 2020).

Social determinants of health such as limited access to affordable health U.S. Immigration and Citizenship Services, 2020) or child care, language barriers, challenges navigating health-care systems, limited health literacy, discordant beliefs about health and illness, profound distrust of the health-care system, gender and ethnocultural incongruence between patients and providers, experiences of discrimination, microaggressions and implicit bias, as well as providers’ lack of knowledge, confidence and cultural sensitivity in caring for FGC-affected populations, further compound distrust and fear due to the political environment (Dawson, Homer, Turkmani, Black, & Varol, 2015; Degni, Suominen, Essén, El Ansari, & Vehviläinen-Julkunen, 2012; Essén et al., 2000, 2011; Evans et al., 2019b; Herrel et al., 2004; Johnsdotter, 2019; Johnson, Reed, & Batra, 2005; Lane et al., 2019; Lazar et al., 2013; Moxey & Jones, 2016; Pavlish, Noor, & Brandt, 2010; Relph, Inamdar, Singh, & Yoong, 2012; Smith & Stein, 2017; Sureshkumar et al., 2016; Tracy, 2007; Turkamani et al., 2019). A cross-sectional study in Oslo, Norway, found that only 20% of Somali migrants sought health care for FGC-related concerns (Mbanya, Gele, Diaz, & Kumar, 2018). Such profound distrust and shortfalls in health-seeking behavior ultimately manifests in disproportionate health disparities and adverse health outcomes (Degni, Suominen, Essén, El Ansari, & Vehviläinen-Julkunen, 2011; Essén, Binder, & Johnsdotter, 2011; Herrel et al., 2004).

## Practical Considerations for Clinicians and Researchers

Before culturally informed discussions encompassing FGC, as shown in Table 1 of the Brady et al.’s (2019) Target Article, can ensue, health-care providers must first comprehend the complex political, historical, sociocultural, and societal contexts in which these discussions are taking place, and how they are embodied. Only then can the framework of safety, respect, and trust between the patient and provider begin to be established, and progress made toward shared decision making between providers and patients, alongside concomitant enhancement in providers’ clinical, counseling and surgical skills.

**Table 1:** Practical considerations for clinicians and researchers when engaging with FGC-affected communities

Action Items	Clinicians	Researchers	Community engagement
Establish trust with FGC-affected communities	X	X	X
Promote equitable community partnerships through community-based participatory research (CBPR) that employs mixed methods		X	X
Develop linguistic and cross-cultural equivalency in the development and use of validated instruments		X	X
Facilitate intergenerational and mixed-gender dialogue across youth, elders, religious and community leaders to engender mutual understanding within communities and support networks that mitigate acculturative processes in the embodiment of FGC in the west		X	X
Explore how FGC has affected patients and their families (whether positively and/or negatively) and provide education and counseling in a nonjudgmental and non-stigmatizing manner	X	X	X
Partner with multi-lingual cultural brokers such as cultural health/peer navigators to enhance community trust and health literacy and facilitate patient navigation of health care	X		X
Optimize the most appropriate timing, route of anesthesia, and location (office-based vs surgical suite) for defibulation procedures based on individualized patient, community, provider, and health-care system-level factors	X	X	X
Enhance competency in the cultural, clinical, and surgical nuances of caring for women with FGC incorporating interactive and enduring educational learning tools (e.g., standardized patient exercises, surgical simulations, and continuing medical education)	X		
Establish referral networks or ‘one-stop-shop’ referral centers comprising multidisciplinary specialists with experience caring for FGC-affected populations, encompassing such arenas of expertise as: social work, psychotherapy, sex therapy, pelvic floor physical therapy, community/peer advocates, and relevant clinicians across medical and surgical subspecialties	X		X
Ensure ethnocultural specificity in data collection methods, track longitudinal outcomes, develop patient morbidity registries, establish quality improvement metrics, and implement patient safety bundles to enhance the safety and quality of clinical care	X	X	
Examine one’s own implicit biases, and cultural/racial power hierarchies in the patient–provider relationship as it pertains to engaging in research, clinical discussions and/or counseling of FGC-affected women, and shared decision-making	X	X	
Encourage the development of training pipelines for health-care professionals representing the ethnocultural and linguistic diversity of populations affected by FGC	X	X	X
Debate the re-classification of “partial” re-infibulation/“partial” defibulation as female genital cosmetic surgery among consenting, autonomous *adult* women with healthy sexual function who request such procedures	X	X	
Consider the larger umbrella of issues that may be of importance to FGC-affected communities, including: economic empowerment, gender equity, intimate partner violence, stigma reduction, etc., which may exert an influence on women’s decision-making	X	X	X
Foster public–private partnerships and multi-center collaborations in the pursuit of funding opportunities	X	X	X

There are health-care system-level factors that are important to consider in order to ensure equitable access to care for women with FGC. Of note, it may be beyond the scope of practice for some family medicine providers and nurse midwives to perform defibulation outside of intrapartum care. Therefore, referral networks are necessary to ensure that patients who opt for antenatal defibulation or defibulation outside of pregnancy are able to access these services. Moreover, referral centers with specialized multidisciplinary expertise should be developed, particularly in regions with scarce resources where providers may lack sufficient volume of exposure to maintain sufficient clinical competencies. Efforts should also be made to honor patients’ requests for intrapartum defibulation even when cesarean delivery is ultimately performed for obstetric indications (Varol et al., 2016). Future research should determine the appropriate timing (Gupta & Latthe, 2018), route of anesthesia, and location (office-based versus surgical operating suites) of defibulation procedures based on individual patient (e.g., concern for triggering post-traumatic distress from flashbacks), provider, and health-care system-level factors (Abdulcadir, Demicheli, Willame, Recordon, & Petignat, 2017). Protocols must be established to optimize accurate clinical documentation using the WHO classification schema, ICD-10 and CPT coding. How these considerations inform women’s decision-making regarding defibulation and/or re-infibulation are yet to be clearly delineated and should be included in future research directives. While certainly not exhaustive, Table 1 provides a list of some best practices that may serve as a guide for clinicians and researchers engaged with FGC-affected communities.

In conclusion, this Commentary sheds light on the dynamic hegemonic context in which patients’ attitudes, norms, and autonomy embodying FGC are being shaped. Besides the ongoing trauma of stigmatization and “othering” upon migration to the west, African women are also thrust into the African-American discourse surrounding historical trauma from centuries of slavery, structural inequities, and racialized stereotypes. Health-care providers and researchers have a responsibility to acknowledge these societal forces and intentionally work to earn the trust of their patients with FGC. Nurturing trust-based, equitable partnerships with FGC-affected communities is a first step toward the development of best practices in optimizing women’s experiences with care and outcomes. It is imperative that health-care providers enhance their competency concerning cultures in which FGC is practiced, medical and surgical care algorithms and protocols (within their scope of practice), as well as seek to engage a multidisciplinary referral network in optimizing care. When counseling women on vulvar reconstructive procedures, providers must compassionately and patiently work with their patients as they proceed through dynamic acculturative processes in facilitating shared decision-making, being mindful of how innate power hierarchies and racial and cultural hegemony may influence the patient–provider relationship. Health care is a human right; as such, it is the responsibility of providers and health-care systems to create environments in which patients feel valued, safe and respected. Cultural, religious, political, historical, social, and hegemonic forces are at play in reshaping the lived experiences of diasporic women with FGC living in the West; we challenge providers and researchers not to propagate the same.

## Footnotes

1 World Art Day in Sweden April 15, 2012. Video featuring male performance artist, Makode Aj Linde, whose blackfaced, minstrel caricature echoes the racialized stereotypes of the post-Civil War era in United States history wherein African-Americans were dehumanized and demeaned in the public media and entertainment with exaggerated facial features, language, movement, character and deportment; codifying blackness as primitive, hypersexual, and comedic. https://www.youtube.com/watch?v=z8zLM_hIw7Y (accessed 2/6/20).

2 Jezebel’s Erin Gloria Ryan. 4/17/12. “Swedish Official Gleefully Cuts Racist Black Lady Cake, Delights Onlookers.” https://jezebel.com/swedish-official-gleefully-cuts-racist-black-lady-cake-5902672 (accessed 2/6/20).

3 Ebony magazine’s Jonathan Pitts-Wiley. 4/18/12. “Taking the 'Painful Cake': Reconsidering the Swedish Ministry Art Nightmare” http://www.ebony.com/news-views/the-swedish-ministry-art-nightmare#.UyOhZrlOXVi (accessed 2/6/2020).

4 Ebony Magazine’s Jamilah Lemieux. 4/18/2012. “When Art Goes Wrong: Black Women's Pain is Not a Prop” http://www.ebony.com/news-views/black-womens-pain-is-not-a-prop#ixzz2w3Jq0TtI (accessed 2/6/20).

5 “Partial” denotes the retention of the integrity of the superficial scarring of the vulvar skin located above the level of the urethral meatus. In the performance of either “partial” defibulation or “partial” re-infibulation, in adult women with type IIIb FGC who otherwise possess healthy sexual function and autonomy in their desire for vulvar reconstructive surgery; the integrity of the superficial vulvar skin, covering over anatomically intact sexual erectile tissues comprising the clitoral glans and vestibular bulbs, is preserved.
